# The seasonal occupancy and diel behaviour of Antarctic sperm whales revealed by acoustic monitoring

**DOI:** 10.1038/s41598-018-23752-1

**Published:** 2018-04-03

**Authors:** Brian S. Miller, Elanor J. Miller

**Affiliations:** 1 Australian Antarctic Division, Kingston, Tasmania Australia; 2 E Miller Consulting, Hobart, Tasmania Australia

## Abstract

The seasonal occupancy and diel behaviour of sperm whales (*Physeter macrocephalus*) was investigated using data from long-term acoustic recorders deployed off east Antarctica. An automated method for investigating acoustic presence of sperm whales was developed, characterised, and applied to multi-year acoustic datasets at three locations. Instead of focusing on the acoustic properties of detected clicks, the method relied solely on the inter-click-interval (ICI) for determining presence within an hour-long recording. Parameters for our classifier were informed by knowledge of typical vocal behaviour of sperm whales. Sperm whales were detected predominantly from Dec-Feb, occasionally in Nov, Mar, Apr, and May, but never in the Austral winter or early spring months. Ice cover was found to have a statistically significant negative effect on sperm whale presence. In ice-free months sperm whales were detected more often during daylight hours and were seldom detected at night, and this effect was also statistically significant. Seasonal presence at the three east Antarctic recording sites were in accord with what has been inferred from 20th century whale catches off western Antarctica and from stomach contents of whales caught off South Africa.

## Introduction

Sperm whales worldwide were commercially hunted from the late 1700 s up until the mid-late 1980s when the International Whaling Commission’s ‘moratorium’ on commercial whaling went into effect. In the Antarctic, sperm whales were hunted from the early 20^th^ century until 1979, though those taken were almost exclusively mature males that are believed to make long migrations between the Antarctic and the tropics. The abundance of some populations of sperm whales in the Southern hemisphere was known to have been reduced considerably during whaling^1,2^. The expected rate of increase for exploited populations of sperm whales was estimated to be approximately 1.1 percent per year^3^. Yet, heavily exploited populations of sperm whales in the Southern Hemisphere have shown little evidence of population increase decades after the end of their commercial hunting^1,4,5^.

Sperm whales are sexually dimorphic and have stratified distributions with males growing larger and venturing to higher latitudes than females. Females are rarely found outside of the subtropics, while males make long migrations between the tropics and high-latitudes and can regularly be found at the edge of polar ice in both hemispheres^6,7^. Group sizes of sperm whales are believed to decrease with increasing latitude; large groups of females and juveniles are found in the tropics; small groups of sub-adult and mature males are found at temperate latitudes, and only lone mature males are found at high latitudes in the Arctic and Antarctic^7^.

Despite nearly a century of whaling, the spatio-temporal distribution of sperm whales in the Antarctic remains poorly described. Peaks in catches of sperm whales at South Georgia and South Shetland whaling stations in December and March suggest a summer migration to high Antarctic latitudes and a return to the sub-Antarctic and subtropics in autumn^8^. Investigation of stomach contents of large male sperm whales caught off Durban, South Africa have led researchers to infer that large male sperm whales return from the Antarctic from May through September^9^.

Knowledge of the occupancy and behaviour of Antarctic sperm whales is fundamental to understanding the sperm whale’s role in the Antarctic ecosystem. In addition to cephalopod beaks, toothfish remains have been found in the stomach contents of sperm whales caught in the Antarctic^9^. Sperm whales have been reported to depredate Patagonian toothfish (*Dissostichus eleginoides*) from long-lines in the sub-Antarctic^10–12^, but have not been reported to interact with in the smaller exploratory fisheries for Antarctic toothfish (*Dissostichus mawsoni*). In addition to serving as a potential baseline and point of comparison with the sub-Antarctic, improved data on seasonal occupancy of Antarctic sperm whales may be especially relevant to marine environmental managers in the Antarctic where a precautionary Ecosystem Based Approach is applied via the international Convention on the Conservation of Antarctic Marine Living Resources^13^.

## Acoustic behaviour

Sperm whales spend a large proportion of their time foraging deep underwater, and during most of this time they produce loud impulsive vocalisations, (henceforth referred to as clicks)^14–18^. Sperm whale clicks are typically classified into four categories based on vocalisation rate (inter-click interval; ICI): slow clicks, usual clicks, creaks, and codas. Slow clicks and codas are believed to be linked to communication^19,20^, while usual clicks and creaks are strongly linked with echolocation and foraging^21,22^. Thus, the form, function, and rate of sperm whale clicks has been reasonably well described, for both males and females and for a number of different populations worldwide.

Usual clicks, named because they are the most commonly detected vocalisation from sperm whales, are highly directional and very intense with apparent source levels measured in excess of 230 dB re 1 μPa peak-peak at 1 m^23,24^. Despite their highly directional beam pattern (roughly 6–20 dB below maximum at 20° off-axis)^23,25^, usual clicks are often detected up to 10–20 kilometres away using a wide variety of underwater recording equipment and detection algorithms^26–35^. Usual clicks are produced at rates of 0.5–2 times per second throughout 80% of a foraging dive^14–16^.

## Long-term acoustic monitoring

The propensity for sperm whales to persistently and repeatedly make loud clicks makes them highly amenable to passive acoustic observations^36,37^. Prior acoustic studies of sperm whales in the Antarctic have made use of towed arrays of hydrophones^34^, however advances in autonomous recording devices have now made it practical to record continuously for long periods of time at sampling rates sufficient to distinguish echolocation clicks of sperm whales^38–41^.

Diel patterns of sperm whale vocalisations have been reported in only a handful of other publications^39–41^. When taken together, these studies suggest considerable variability across both locations and time of year. A study of sperm whales in the Mediterranean found that they were more likely to detect sperm whales during daylight hours than night^40^. However, this is the opposite of what was reported off Hawaii where detections of sperm whale clicks were significantly more likely to occur during the night^39^. A separate study of sperm whales in the Ligurian sea, indicated the distribution of detections of sperm whales shifted from day to night in September i.e. autumn in the northern hemisphere^41^. Tagging studies also reveal variable results regarding diel patterns in sperm whale behaviour. A study of five sperm whales tagged in the Gulf of California in November found that they dive to “somewhat shallower depths” at night, though not to the same degree as that of their main prey species in the area, Humboldt squid^42^. Yet, a more recent analysis of the dive and location data from 26 sperm whales tagged in March and April in the Gulf of California found no evidence of diel changes in diving behaviour^43^.

**Figure 1:**
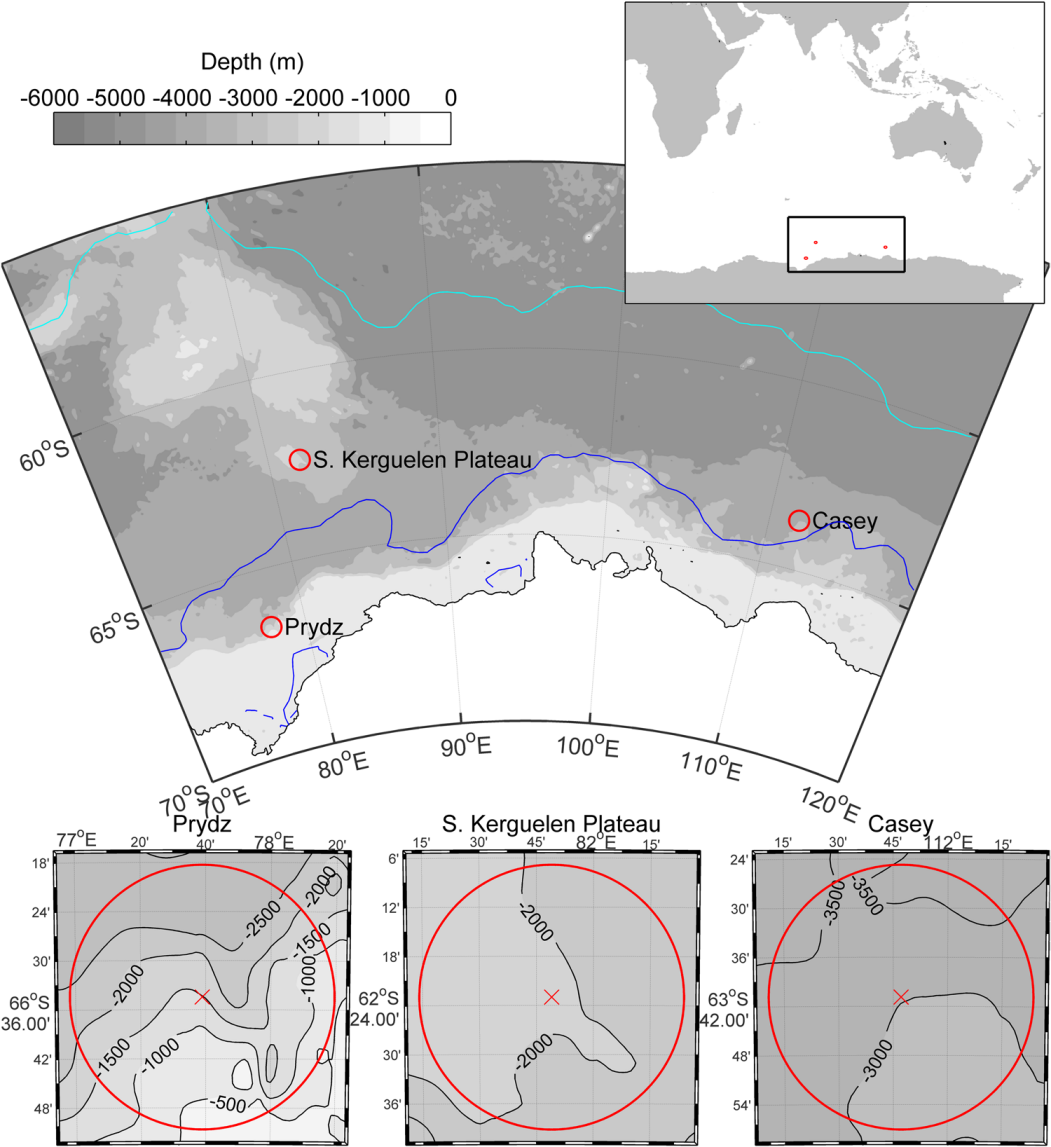
Map of location of long-term recording sites used in this study. Crosses mark the nominal deployment location. Red circles indicate a 30 km radius around each recording site. The edge of the sea ice for 2014 is plotted for February 01 (dark blue line) and September 01 (light blue line) to provide an indication of summer minimum and winter maximum sea-ice extent. Map created using M_Map version 1.4 h and ETOPO 1 bathymetry/altimetry^77^ (https://www.eoas.ubc.ca/~rich/map.html). Sea ice edge was extracted from data the National Snow and Ice Data Center^78^ using the Matlab package Antarctic Mapping Tools^79^.

Here we use data from moored autonomous acoustic recorders to investigate the acoustic presence of sperm whales at three Southern Ocean locations off East Antarctica (Fig. 1). We focus on the detecting usual clicks, and we describe a simple automated classification scheme based on ICI to determine whether sperm whales are acoustically present in a given hour of recorded audio. We then apply this method to a long-term dataset to conduct the first acoustic investigation of the seasonal occupancy and diel behaviour of sperm whales in the Antarctic.

## Results

### Classifier performance and characterisation

**Figure 2:**
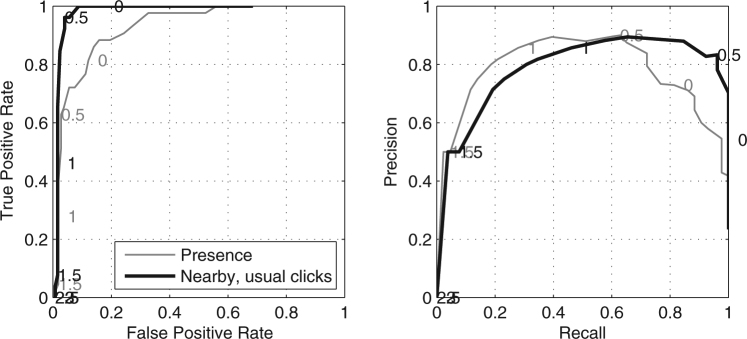
Receiver-operator characteristic (ROC) and precision-recall (PR) curves for our classifier using the 150 hours of “ground-truth” data. The grey line shows ROC & PR using manually assessed presence as the ground-truth (43/150 hours with whales present). The black line shows the ROC using only ground truth recordings containing ‘usual clicks’ from ‘nearby’ whales (26/150 hours with these conditions). Numbers indicate the threshold at each point with S > 300 ICIs per hour for all thresholds (see Methods for detailed description).

Our ICI classifier performed well at detecting the presence of sperm whales, and our 150 hour ground-truth dataset allowed us to characterise and quantify its performance. Area under the receiver-operator characteristic curve (AUROC) for sperm whale presence was 0.928. Our classifier performed even better for ‘nearby’ sperm whales that were producing usual clicks yielding an AUROC of 0.982. At our chosen threshold of T = 0.5, our classifier yielded a true positive rate/recall of 96.2%, false positive rate of 4.0%, and precision of 83.3%, for ‘nearby’ sperm whales for the ground-truth dataset (Fig. 2).

### Seasonal occupancy

Across all three sites and years 46,133 hours of data were analysed yielding 1065 true positive hours with sperm whales after removal of 498 false positive classifications (Table 1). This yielded an overall false positive rate of 1.1%. False positive rates across sites ranged from 0.4% to 3.5%, and all were lower than or similar to that of 4.0% from our ground-truth dataset.

**Table 1:** Confusion matrices for detection and classification of sperm whales at three Antarctic sites. Classifier parameters (described in detail in the text and Equation 1) were T = 0.5 and S >300 ICIs per hour.

**Site**	**Hours Analysed**	**True Positives**	**False Positives**	**False positive rate (%)**
Prydz	6866	62	241	3.5
Casey	13513	359	160	1.2
S. Kerguelen Plateau	25754	644	97	0.4
**Total**	**46133**	**1065**	**498**	**1.1**

**Figure 3:**
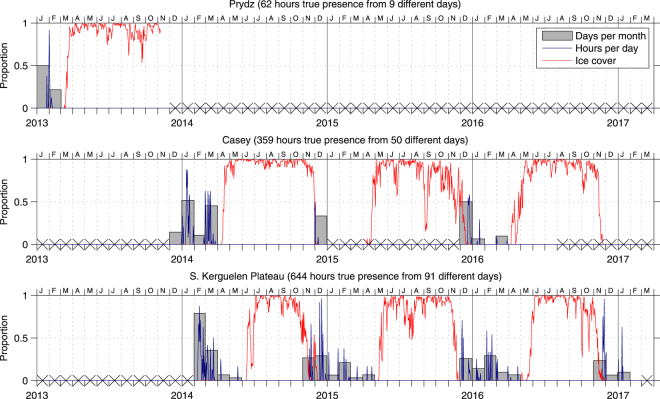
Bars show proportion of effort days per month with acoustic detections of sperm whales. The blue line shows the proportion of hours per day with true positive detections of sperm whales. Tick marks indicate the start of each month. The red line indicates the proportion of ice concentration within a 30 km radius of each recorder from AMSR2 satellite imagery^74^. Crosses on the axis indicate months with no data available^80^.

Sperm whales were detected at all three Antarctic sites in every year that data were collected predominantly in summer months from Dec–Feb (Fig. 3). The probability of sperm whale presence was found to significantly decrease with increasing ice cover (OR: 5.8 × 10^−5^, CI_95%_ 2.4 × 10^−8^-0.002; *p* = 0.0006). No sperm whales were detected during ice heavy winter months (Jun–Aug) or early spring (Sep, Oct). At Casey and South Kerguelen Plateau, sperm whales were detected every March for which data were available. At the South Kerguelen Plateau site whales were detected every April, two of the three Novembers, and once in May. The number of days with true positive detections was variable across months, ranging from 0 to 16. Again, summer months typically had more days with true positive detections than spring or autumn.

### Diel behaviour

**Figure 4:**
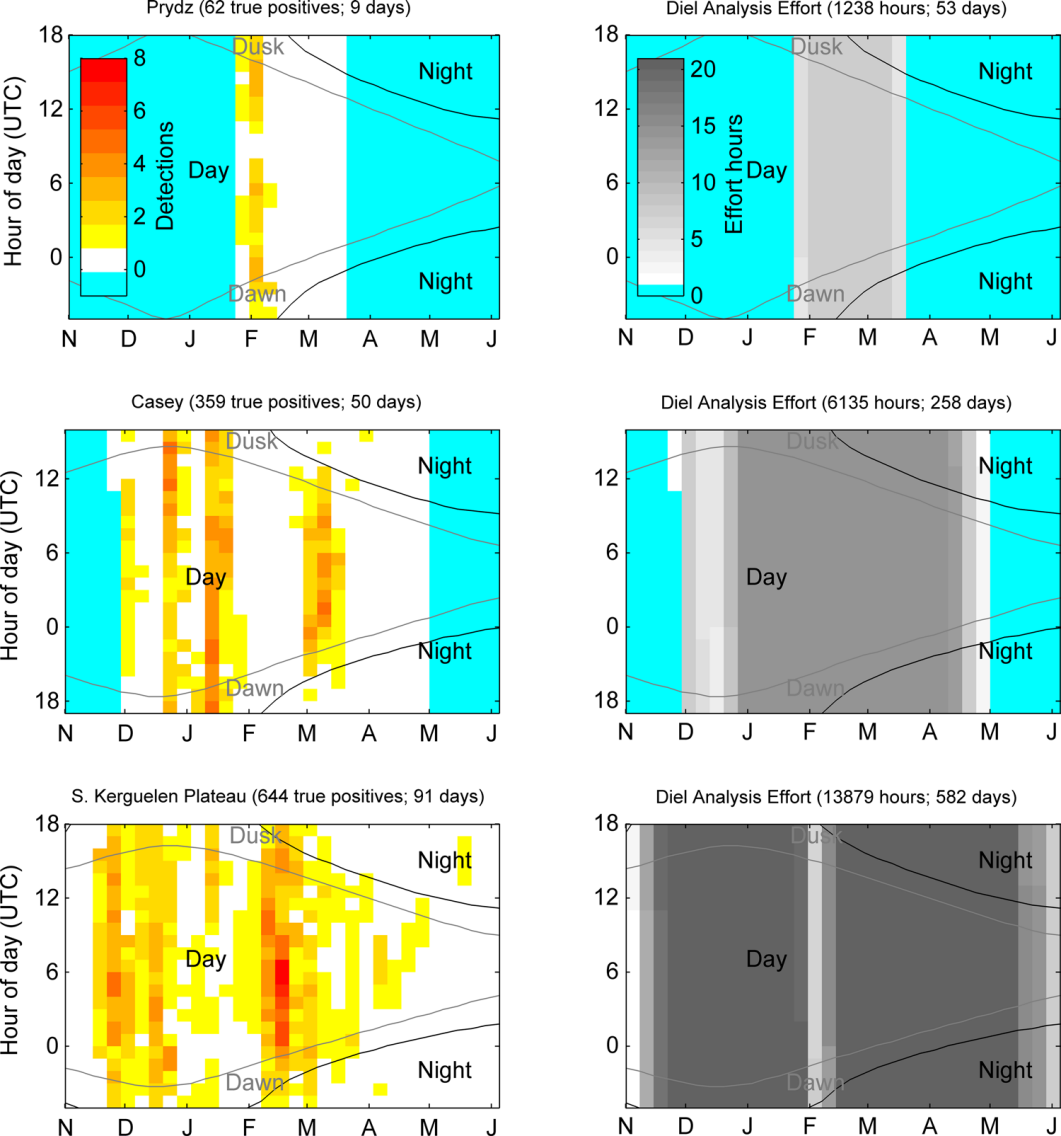
Detections of sperm whales (left column) and hours of effort used for the diel analysis (right column) as a function of day of year and hour of day for each site. In this 2D histogram, all years of data have been aggregated into 7-day time bins. The Dawn and Dusk labels indicate periods of nautical twilight. Blue shading indicates time periods that were not included in the diel analysis from either lack of recordings (Nov-Jan at Prydz) or ice exclusions (all other excluded months). Months June through October are neither shown nor included in the diel analysis since no detections of sperm whales were made and the sites were fully ice covered during these months.

Visualisation of the hours with true positive detections indicated that sperm whales in the Antarctic click almost exclusively during the day and nautical twilight (Fig. 4; Table 2). In months with only nautical twilight and no ‘true’ night (November, to early February) sperm whales were detected at all hours of the day with maximums of 22, 21, and 23 hours per day at Prydz, Casey, and Kerguelen respectively (Fig. 4). In months with ‘true’ night (mid-February to May), sperm whales were only detected at Casey and South Kerguelen Plateau sites, with maximums of 15, and 17 hours per day respectively and hardly any detections during night-time hours (Fig. 4).

**Table 2:** Effort and true detections for diel analysis by site and light regime. Odds-ratio, its 95% confidence intervals (CI), and the p-value of the GLM parameter estimates are all in comparison to Day for each site.

Site	Light regime	Ice-free effort (hours)	True positive (hours)	% true	Odds-ratio	Odds-ratio 95% CI	GLM p-value
Prydz	Day	808	46	5.7	—	—	—
	Dusk	159	8	5.0	0.88	0.38–1.80	0.74
	Night	132	0	0.0	1.4 × 10^−7^	0–1012	0.98
	Dawn	139	8	5.8	1.01	0.43–2.08	0.98
Casey	Day	3916	290	7.4	—	—	—
	Dusk	585	28	4.8	0.63	0.41–0.92	0.02
	Night	1024	7	0.7	0.86	0.04–0.17	1.7 × 10^−10^
	Dawn	610	34	5.6	0.74	0.50–1.05	0.10
S. Kerguelen Plateau	Day	8079	515	6.4	—	—	—
	Dusk	1345	50	3.7	0.57	0.42–0.75	0.0002
	Night	3139	29	0.9	0.14	0.09–0.20	<2 × 10^−16^
	Dawn	1316	50	3.8	0.58	0.43–0.77	0.0003

After excluding time periods with heavy ice cover, recording effort was not even across all light regimes, with more effort in Day than in Dawn, Dusk and night combined. Nevertheless, light regime was found to significantly affect sperm whale presence at both the Casey and Kerguelen sites, but not at Prydz (Table 2). At Casey, the odds of sperm whale presence during Day was 37% higher than Dusk (CI_95_ 8–59%), and 91% higher than at Night (CI_95_ 83–96%). At Kerguelen, Day had significantly higher chance of sperm whale presence than all other light regimes (Table 2). Specifically, the odds of sperm whale presence during Day was 43% higher than Dusk (CI_95_ 25–58%); 86% higher than Night (CI_95_ 80–91%); and 42% higher than Dawn (CI_95_ 23–57%).

## Discussion

Our simple classifier performed well on our Antarctic dataset with our chosen threshold. The high true positive rate of 96% of ‘nearby’ sperm whales from the ground-truth dataset gave us confidence that we had adequately captured a high proportion of the hours when sperm whales were present and vocalising in the full dataset. The low false positive rate of our ICI classifier greatly reduced the amount of manual inspection required to remove false positives from the results. Additionally, the relatively low number of hours with sperm whales present (1,065/46,133) further expedited verification of true positives.

The precision of our classifier for the full dataset was 0.68 (i.e. 32% of detections were false positives). The precision of our detector on the full dataset was lower than that of the ground-truth dataset which was 0.83 at the same threshold (Fig. 2). This difference in precision likely arises from the greater imbalance between positive and negative classes in the full dataset compared to that of the ground-truth dataset (1,065/45,068 vs 26/124 respectively). Nonetheless, the precision of the classifier on the full dataset was acceptable since the total number of true and false positive classes was still small enough to allow for manual inspection of all positive classifications in a reasonable timeframe.

Several factors contributed to the success of our analytical methods. First, the vocal behaviour of sperm whales in the Antarctic appears consistent with that reported for other high-latitude populations i.e. animals predominantly producing usual clicks, often for many hours per day^14,38,44^. Second, in the Antarctic there are very few other sound sources that produce clicks at the same ICI as sperm whales. When reviewing all of the detections and inspecting our ‘training’ data, we did occasionally notice hours where multiple whales were detected. Though we did not attempt to quantify the total number of hours with multiple whales or the number of whales per hour, our classifier was still able to correctly identify these hours as true positive detections. We suspect that this outcome was due in part to the presence of sufficient timespans within these hours when only one whale was clicking, and thus sufficient number of ICIs in the ‘sperm whale range’ were generated to trigger a detection.

The noise source for the bulk of the 498 false positive classifications was impulsive ice sounds with most false positives occurring in winter and early spring months. Similar to recordings from the Gulf of Alaska^38^, we found humpback whale vocalisations triggered false positives for our classifier on one or two occasions. The bioduck call of Antarctic minke whales^45,46^ also triggered false positives for our classifier on a very small number of occasions, always in winter. For the Prydz dataset, a relatively narrowband, unidentified, impulsive noise source was responsible for most of the false positive detections and the notable difference in false-positive rate between Prydz and the other sites.

Similar to results from the Gulf of Alaska, we found a summer peak in acoustic detections of sperm whales^38^. In summer, the proportion of detection-days per month in our study was also similar to that reported for the Gulf of Alaska. However, unlike the Gulf of Alaska, none of our Antarctic sites yielded year-round detections of sperm whales.

The absence of winter and early spring detections correlated significantly with ice cover (p < 0.001; Fig. 3). All of our recording sites were fully covered by more than 30 km of sea ice throughout winter (Fig. 1). Unlike blue and fin whales, whose calls can propagate underneath sea-ice and travel over hundreds or even thousands of kilometres with minimal attenuation^47–49^, the measured and modelled effective detection range of the usual clicks of sperm whales is on the order of 20–30 km^34,35,50^, though this will vary by instrument and recording environment. Furthermore, noise levels at our Antarctic sites were quietest in winter so we would expect to more readily detect any echolocating sperm whales if present. Unlike baleen whales, sperm whales are income-breeders^51^ and forage (and presumably echolocate) throughout the year. Thus, the lack of winter detections of sperm whales suggests that sperm whales were simply not present in the heavy ice surrounding our recorders. However, further data collected from north of the winter ice edge would be required to better answer the question of whether sperm whales are still present in ice-free Antarctic waters during Austral winter.

Over hourly and monthly time scales sperm whales have very well understood and consistent vocal behaviour, and the properties and detection range of their usual clicks have been extensively described^21,23,34,35,50,52–54^. Their near-constant echolocation during deep dives is indicative of regular and efficient foraging throughout oceans and all throughout the year^16,33,38–40^. Thus, they may be one of the few cetaceans where it could be relatively safe to interpret a lack of detections of vocalisations as an actual absence of animals – at least over time periods longer than a few days.

However, when considering sperm whale vocal behaviour over daily timescales, the small number of studies of diel behaviour reveal considerable variability across locations and time of year^39–43^. Our observations of a significantly higher likelihood of daytime detections at our Casey and S. Kerguelen Plateau sites are consistent with results reported on a study of sperm whales from the Mediterranean^40^; the opposite of what was reported off Hawaii where detections of sperm whale clicks were significantly more likely to occur during the night^39^; and different from the shift in the distribution of detections from day to night in autumn in the Ligurian sea^41^. The lack of significant diel patterns at our Prydz site could be in accord with the aforementioned site variability among other studies. However we believe the lack of significant diel pattern at this site is more likely driven by the smaller sampling effort at that site, which in turn resulted in a very small number of true positive detections that yielded less statistical power than the other two sites.

A plausible, if not simplistic, explanation for the lack of night detections is that sperm whales rest or sleep at night in the Antarctic. There have been very few studies of the resting behaviour of wild cetaceans, but suction-cup tag data has revealed that rest comprised 7.1% of the time budget of 59 sperm whales tagged throughout the Northern hemisphere^17^. In our study, night-time effort comprised 25% of ice-free hours, which suggests that if sperm whales are indeed resting at night when there are no detections, the resting behaviour of sperm whales in the Antarctic could be very different than in the mid-latitudes of the northern hemisphere.

The usual clicks of sperm whales are strongly linked to foraging behaviour^21,22^, so we, like others who have detected diel trends in sperm behaviour^39,40,42^ suggest this lack of night-time detections is potentially driven by changes in prey behaviour. Night time rest could be an effective energy-saving strategy if prey were less available at night (e.g. capture of prey required expenditure of more energy at night than in the daytime). The top four species of squid found in the stomachs of Antarctic sperm whales during industrial whaling were *Kondakovia longimana* (giant squid), *Mesonychoteuthis hamiltoni* (colossal squid), *Gonatus antarcticus*, and *Moreteuthis knipovitchi*, and these four species comprised 99% of the stomach contents by weight^9^. Three of these four squid species are known to prey upon Antarctic krill^55^, which are in turn known to show strong diel changes in their behaviour^56^. Thus, it is plausible that diel changes in sperm whale behaviour might be linked to diel changes in prey behaviour and availability.

Unfortunately, our near-total absence of night detections and *in-situ* observations of prey provide us with little hard evidence as to the exact cause of the observed diel pattern in vocal behaviour. Further studies using time-depth recorders^42,43^ or suction-cup archival tags^16,57,58^ could shed metaphorical and/or literal light on exactly how sperm whales spend their short, autumn, Antarctic nights. Further long-term acoustic recordings at ice-free Antarctic and sub-Antarctic latitudes may also provide additional information on winter and spring occupancy and diel behaviour of sperm whales.

The results of our study may also be considered a precursor to an acoustic estimate of population density of sperm whales in the Antarctic. Considerable additional analysis would be required to convert our raw acoustic data into a density estimate (e.g. whales/km^2^/h). Provided that diurnal patterns can be better quantified and explained, the otherwise well-known, consistent, and predictable behaviour of sperm whales in the Antarctic should facilitate estimation of the required cue rates^14^, detection probabilities & distances^50,59,60^, and multipliers required for density estimation^35,61^. Acoustic density estimates could then be compared with historical densities from the IDCR-SOWER visual and acoustic surveys^5,62^, or with historical densities from catches recorded by southern ocean whalers^63^. In addition to population density estimated from usual clicks, estimates of sperm whale creak/buzz rates could potentially yield more detailed information on feeding rates since creaks/buzzes are known to indicate the final phase of foraging^21^.

Furthermore, investigation of acoustic properties of clicks may also provide estimates of the size distribution of Antarctic sperm whales. The relationships between the fine-scale acoustic pulse structure of clicks and length of the whale are reasonably well known^52,64–66^, and methods for acoustic size estimation can be readily automated^67,68^.

## Conclusions

We have created an automated method for assessing the temporal presence of sperm whales in the vicinity of long-term acoustic recording sites. The method is easy to apply, fast to compute, reliable, and thus provides a means to efficiently characterise the seasonal presence of sperm whales. Application of this method to three Antarctic recording sites has revealed new insight into the seasonal presence of sperm whales off east Antarctica and provides a modern baseline for the seasonal acoustic presence of sperm whales in the Antarctic.

Sperm whales were detected every year at all three of our recording sites off east Antarctica. Ice was found to have a significant negative effect on sperm whale presence, and as a result, most detections occurred in summer, and no detections were found in winter or early spring. Light regime was found to significantly affect sperm whale presence with a higher likelihood of detections occurring during daylight hours, indicating that in the Antarctic sperm whales change their behaviour at twilight and night. Further analysis of the acoustic recordings presented in this study are likely to yield considerable additional information on the ecology and behaviour of this potentially important, but not well studied, top Antarctic predator.

## Methods

### Data collection

Acoustic data for our study were collected via custom autonomous long-term recording devices that were moored in the Southern Ocean. The recorders were designed and manufactured at the Australian Antarctic Division (Kingston, Tasmania) to operate for year-long, deep-water, Antarctic deployments. They included a factory calibrated HTI 90-U hydrophone (nominal sensitivity of −165 dB re 1 V/µPa and flat frequency response from 2 Hz to 20 kHz) and workshop-calibrated frontend electronics (hydrophone preamplifier, filters, & analog-digital converter). The preamplifier provided a gain of 20 dB, and input was AC coupled with a nominal corner frequency (−3 dB point) of 6.6 Hz. A 6th order lowpass Butterworth filter with a corner frequency (−3 dB point) of 4 kHz and rolloff of 120 dB/decade served as the anti-aliasing filter. The analog-digital converter, based on an AD7683B chip, provided 100 dB of spurious free dynamic range, and a total signal-to-noise and distortion of 86 dB which yielded 14 effective bits of dynamic range at a 1 kHz input frequency. The target noise floor of each recorder was below that expected for a quiet ocean at sea state zero. Electronics were placed in a glass instrumentation sphere rated to a depth of 6000 m, and the sphere was attached to a short mooring with nylon straps to decouple recorder and hydrophone from sea-bed. The hydrophone was mounted above the glass sphere with elastic connections to the mooring frame to reduce mechanical self-noise from movement of the hydrophone.

Recordings were made at three locations in the Southern Ocean off East Antarctica (Fig. 1). All three sites were located along the resupply route to Australia’s Antarctic stations, and these recording sites comprise the eastern Antarctic locations of the Southern Ocean Hydrophone Network^69^. Incidentally, the locations of our recorders were within the boundaries of exploratory fisheries for Antarctic toothfish (Commission for the Conservation of Antarctic Marine Living Resources statistical reporting areas 58.4.1, 58.4.2, 58.4.3b)^70^.

Sites Casey and Prydz were located on the Antarctic continental slope, while the South Kerguelen Plateau site was located at the extreme southern end of the Kerguelen Plateau (Fig. 1). The Prydz site was in operation from Jan-Nov 2013 (6866 total hours). The Casey site yielded underwater acoustic data continuously from in Dec 2014-Dec 2015 and Dec –Jul 2016 (13513 total hours). The South Kerguelen Plateau site yielded data from Feb 2014–Feb 2017 (25754 total hours) with only small gaps of a few days when recorders were replaced each February (Table 3). Recorders operated continuously throughout each annual deployment at a sampling rate of 12 kHz.

**Table 3:** Locations and recording times of acoustic data used in this study.

Deployment name	Latitude	Longitude	Depth	Start date	End date
Casey2014	−63.7955	111.7871	2700	2013/12/25	2014/12/11
Casey2016	−63.8076	111.7361	2700	2015/12/16	2016/07/16
Prydz2013	−66.5747	77.65015	1800	2013/01/26	2013/11/08
Kerguelen2014	−62.3801	81.7968	2000	2014/02/10	2015/02/06
Kerguelen2015	−62.3803	81.7925	2000	2015/02/10	2016/01/29
Kerguelen2016	−62.3696	81.6955	1800	2016/02/06	2017/02/04

### Data availability

Acoustic data used in this study are publicly available via the Australian Antarctic Data Centre http://data.aad.gov.au/metadata/records/AAS_4102_longTermAcousticRecordings Miller *et al*., 2017).

### Analysis

#### Click Detection

Clicks present in the recordings were detected using the click detector module in PAMGuard (version 1.5.11; http://www.pamguard.org)^71^. A full description of the algorithm used by this detector is available via documentation that is distributed with PAMGuard. In short, the Click Detector module operates in a manner similar to a band-filtered energy detector (i.e. a time-domain version of Page’s Test)^72^. Variants of this algorithm have been used in prior studies and software to detect sperm whale clicks^23,73^. The default parameters for the PAMGuard click detector were used as they were found to be highly suitable for detecting the clicks of sperm whales (Table 4).

**Table 4:** Parameters used to detect clicks via the Click Detector module in PAMGuard.

Parameter	Value
Threshold	10 dB
Long filter	0.00001000
Long filter 2	0.00000100
Short filter	0.10000000
Min click separation (samples)	100
Max click length (samples)	1024
Digital pre-filter	Lowpass 4^th^ order Butterworth with corner at 1000 Hz

The click detector in PAMGuard is a general purpose transient detector and will detect many other impulsive sounds in addition to the clicks of sperm whales. Additional impulsive sounds that were commonly noted during aural inspection of our recordings include ambient noise linked to wind; the formation, breakup, and collisions of ice; and short-onset broadband sounds produced by other marine mammals such as Antarctic minke whales, humpback whales, crabeater seals and leopard seals. Due to the remote Antarctic location of recorders, impulsive noise from ships and seismic airguns were seldom present during manual inspections of audio.

#### Click classification

**Figure 5:**
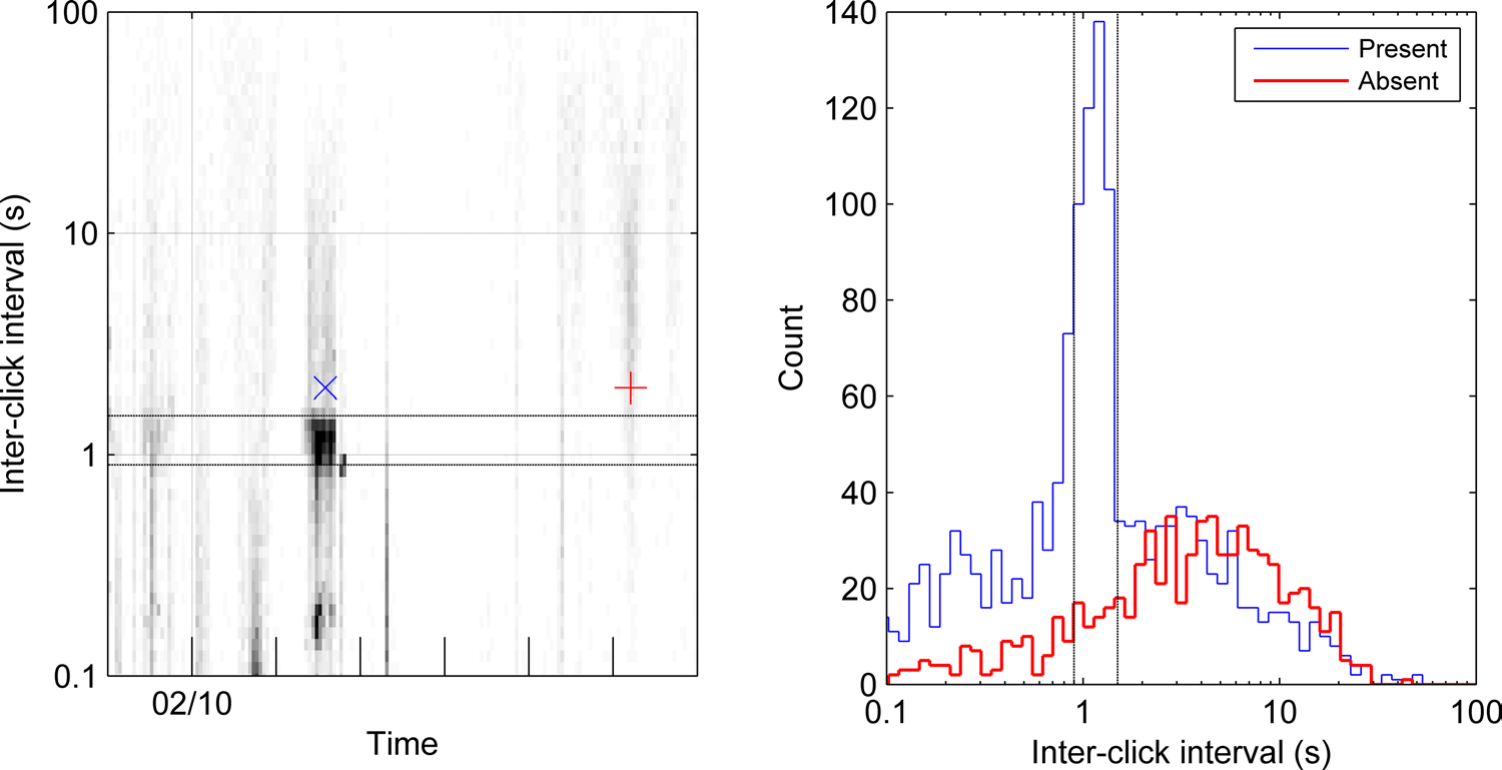
Left: Example ICIgram from a week of data starting on 9 Feb 2013 for the Prydz site. The blue cross marks a time period with manually verified presence of sperm whales. The red plus indicates a time period where the analyst did not detect sperm whales. Right: ICI histograms for two different hour timespans from the Prydz site. The blue line represents the hour long timespan of the blue cross on the left plot (14:00 on 11 Feb 2013). The red line shows an hour-long timespan at the time of the red plus on the left plot (05:00 on 15 Feb 2013). Dashed lines indicate the limits of the ICIs used for classification of sperm whales (bins that comprise S from Eq. 1).

No attempt was made to classify individual clicks or measure acoustic parameters such as intensity, peak-frequency, or bandwidth. Instead, we developed a measure of sperm whale presence based solely on ICI. For a discrete span of time (i.e. 1 hour) we calculated the ICI of all detections. A measure of the distribution of ICIs within that timespan was obtained by accumulating ICIs into a histogram with discrete bins. The bins of the histogram spanned 0.083–3600 s and were logarithmically spaced (Fig. 5). An ICI value of 0.083 was the smallest possible ICI given our parameters for the click detector, and an ICI of 3600 is the maximum possible ICI between two clicks that occur in the same hour.

The time series of ICI histograms were then displayed sequentially to generate a surface plot in order to obtain a synoptic view of the binned ICIs throughout the recording (Fig. 5). We refer to this surface plot as the ICIgram since it is an agglomeration of ICI histograms, and looks somewhat similar to a spectrogram (Fig. 5).

Initial analysis efforts involved manual inspection of the ICIgram and the application of simple heuristics to the ICI histograms. Heuristics were essentially assumptions regarding the typical vocal behaviour of sperm whales, and were based on values reported in the scientific literature and the authors’ own experience. Prior studies have indicated that the usual clicks of sperm whales have mean ICI between 0.9–1.5 s^14,15,73^. Since male sperm whales at high latitude are typically not in very large groups^5^, we simply assumed clicks with ICIs between 0.9 and 1.5 s could potentially belong to sperm whales, and denote the total number of these clicks as *S*. We assigned clicks with ICIs between 3 and 3600 s as non-sperm whale or noise, and denote them as *N*. For each timespan we calculated the base 10 logarithm of the ratio of *S* to *N*, to create a classification function, *C*, which can be written as:

1 $$C={\mathrm{log}}_{10}\frac{S}{N}\{\begin{array}{cc}C\ge T & Sperm\,Whale\\ C < T & Noise\end{array}$$

A classification threshold, *T*, was then applied to distinguish whether a recording contained sperm whale clicks. To further reduce false positives and refine our criteria for presence we only considered sperm whales present when S > 300 for a given hour. In practical terms, including the criteria of S > 300 can be thought of as a requirement that a single whale is producing usual clicks for at least five minutes throughout the hour. This parameter was determined heuristically from initial inspection of true positive hours with sperm whales and false positive hours with impulsive noise from sea-ice. The main purpose of including this additional criteria for detection of S > 300 was to reduce the amount of false positives from sea-ice, which can often produce impulsive sounds at rates similar to that of sperm whales, though typically not more frequently than 300 times per hour. Additionally, this served to limit our definition of presence to time periods when sperm whales were unambiguously near the receiver.

#### Ground truth data and classifier performance

150 hours of data were visually and aurally inspected by an expert (author EJM) to ascertain the presence of sperm whale clicks. The 150 hours were chosen from the recordings made in 2014 at S Kerguelen Plateau and Casey sites by stratifying the hours for each site into 3 groups and randomly sampling 25 hours from each group. Our classification function, *C* was used to stratify the groups as follows: group 1 was intended to capture hours that we believed were likely to have sperm whales (*C* ≥ 1); group 2 was meant to include hours that we believed were not likely to contain usual clicks of sperm whales (*C* ≤ 0); group 3 was intended to capture cases where the threshold for the classifier was in-between groups 1 and 2 (0 < *C* < 1). Manual inspection was blind to stratification group, however detections from PAMGuard’s click detector were overlaid on the spectrogram during inspection.

For each hour inspected, the presence or absence of sperm whale sounds was noted along with the type of sound (i.e. usual clicks and/or slow clicks and/or creaks). For hours with sperm whales present, a qualitative estimate of the proximity of the whales was also recorded. Whales were considered ‘nearby’ when clicks were either intense or detected for a large proportion of the hour. Whales were considered ‘distant’ when clicks were very quiet or only present for a small portion of the hour (e.g. a single short click train or present for just a few minutes).

To assess the performance of our classifier, two receiver-operator characteristic (ROC) and two precision-recall (PR) curves were calculated using the manually inspected classifications as the ground-truth. Presence, as reported by manual inspection, was used as the ground truth-positive class when calculating the first ROC & PR curves. For the second ROC & PR curves, only recordings with ‘nearby’ whales producing usual clicks were used as the ground truth-positive. We report the area under the ROC curve (AUROC) as a measure of the performance of the classifier. An AUROC of 1 indicates the classifier is perfect, while an AUROC of 0.5 indicates that a binary classifier is, on average, no better than chance.

#### Automated detection and classification

After characterising the performance of our classifier, we then applied it to all of the data at all three sites. A threshold of *T* = 0.5 was chosen as a compromise between high true positive rate and low false positive rate (96.2% and 4.0% respectively; see Results section). The classification function and ICIgram were inspected for each site and year, and all hours with positive detections were looked at by an expert to validate true positives and remove false positives.

Removal of false-positives yielded a time-series of the presence of sperm whales at each site with time resolution of 1 hour. From our time-series we then calculated the number of hours per day and the number of days per month with detections of sperm whales. Daily sea ice concentration was extracted from AMSR2 satellite imagery^74^ (6.25 km resolution) for a 30 km radius surrounding each recorder. The daily mean of the sea ice concentration within the 30 km radius was then overlaid on the time series of detections (Fig. 3). The effect of ice on sperm whale presence was analysed using a generalised linear model (GLM) with a binomial distribution and logit link. In addition to proportion of ice, site was initially included as a fixed factor to assess for any difference between the three sites (Casey, Prydz, S. Kerguelen Plateau). Data were binned into 1 day time periods to reduce the influence of temporal autocorrelation between hourly measurements on subsequent analysis and to match with the finest time resolution of available AMSR2 ice data. This analysis was carried out in R^75^ using the package ‘stats’.

We also investigated whether there were diel trends in detections. Due to the statistically significant inverse relationship between sperm whale presence and ice cover (see Results section), we restricted our analysis of diel trends to time periods when there was no ice cover. Specifically, for each site and year we excluded the time between when ice concentration first and last exceeded 60% for that year. The altitude of the sun at each site was calculated at 1 minute intervals to determine sunrise, sunset, and the start of nautical twilight (i.e. when the sun Altitude is between 0 and −12°) for each site^76^. True positive detections were then grouped by hour of day and day of year to create a 2D histogram of hourly detections. Histogram bins for the day of year spanned 7 days. Curves indicating day, night, dawn, and dusk were then overlaid on the 2D histogram to provide visual indication of the light regime at each site (Fig. 4).

In addition to visualising sperm whale presence as a function of hour of the day and day of the year, we also investigated whether light regime had an effect on true detections. Using the solar altitude calculated above, each non-excluded hour of acoustic data was assigned a light regime of either dawn, day, dusk, or night. The effect of light regime on sperm whale presence was then analysed using a generalised linear model (GLM) with a binomial distribution and light regime (Day, Dusk, Night, Dawn) as a fixed factor. A separate model was run for each of the three sites since diel patterns reported in prior studies suggest variability across widely spaced sites^39–41^. Odds-ratios (OR) and their associated 95% CIs were estimated in the final logistic models to assess associations among light regimes and sperm whale presence. This analysis was carried out in R^75^ using the package ‘stats’.

### Permitting authority

Data used in this manuscript were collected under authorisation of the Australian Antarctic Division, Department of the Environment and Energy in accordance with section 12D of the Antarctic Treaty (Environment Protection) Act 1980 of the Commonwealth of Australia.
